# Diisopropyl 2-[(4-nitro­benzo­yl)amino]­propane­dioate

**DOI:** 10.1107/S2414314623001049

**Published:** 2023-02-14

**Authors:** Cristi P. Deleon, Anthony D. Ramirez, Diana Gonzalez, Arghya Ghosh, László Kürti, Muhammed Yousufuddin

**Affiliations:** a University of North Texas at Dallas, 7400 University Hills Blvd., Dallas, TX 75241, USA; b Rice University, 7400 University Hills Blvd., Houston, TX 77030, USA; Katholieke Universiteit Leuven, Belgium

**Keywords:** crystal structure, 4-nitro­phen­yl, isopropyl ester

## Abstract

The title compound, C_16_H_20_N_2_O_7_, crystallizes in the space group *C*2 with two mol­ecules in the asymmetric unit. The crystal packing shows O⋯π inter­actions between the two mol­ecules.

## Abstract 2

The title compound crystallizes in the space group *C*2 with two mol­ecules in the asymmetric unit.

## Graphical abstract

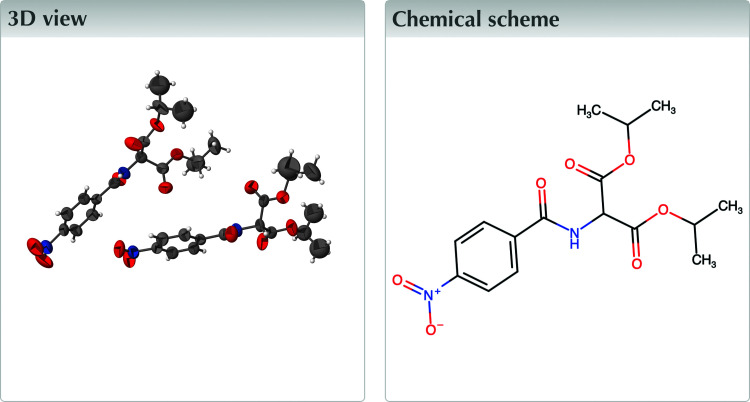

## Structure description

**Figure 1:**
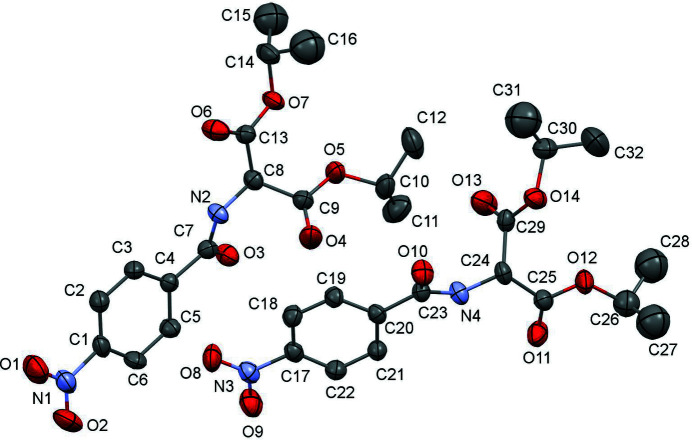
Mol­ecular structure of the title compound with displacement ellipsoids drawn at the 30% probability level. Hydrogen atoms are omitted for clarity.

**Figure 2:**
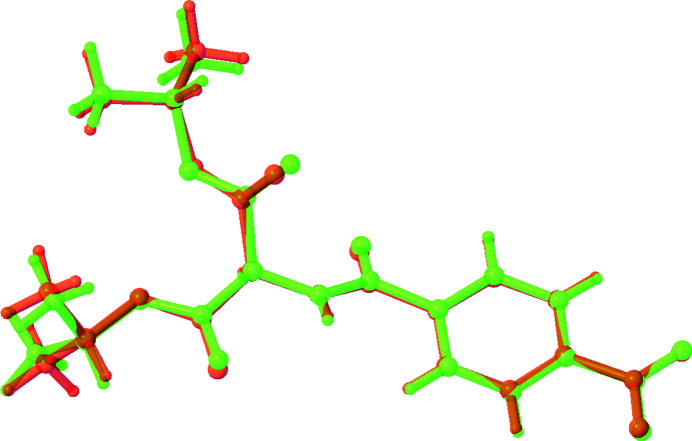
Overlay plot of the two mol­ecules in the asymmetric unit. Mol­ecule *A* is shown in green, mol­ecule *B* in red.

**Figure 3:**
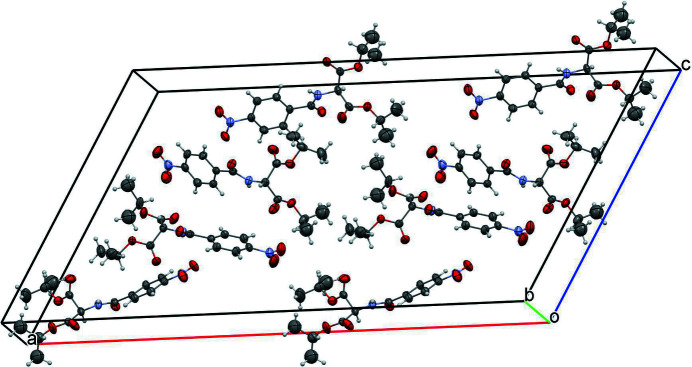
Packing diagram for the title compound.

The title compound [alternative name: diisopropyl 2-(4-nitro­benzamido)­malonate] was made in an attempt to make a compound with an N_3_ cyclic ring. The starting material for this compound has been used previously to make symmetrical and unsymmetrical secondary amines (Kattamuri *et al.*, 2017 ▸). The title compound crystallizes in the monoclinic space group *C*2 with two mol­ecules in the asymmetric unit (*Z* = 8). Mol­ecule *A* contains nitro group N1—O1—O2, and mol­ecule *B* contains nitro group N3—O8—O9 (Fig. 1 ▸). Both nitro groups are almost coplanar with their attached phenyl rings [dihedral angles = 3.7 (11)° for mol­ecule *A* and 3.3 (8)° for mol­ecule *B*]. An overlay of the two mol­ecules indicates an almost complete overlap with only slight deviations in the ester groups (r.m.s. deviation = 0.268 Å; Fig. 2 ▸). The dihedral angle between the phenyl rings is 38.0 (3)°. The closest inter­actions in the packing are observed between the two mol­ecules in the asymmetric unit (Fig. 3 ▸): an N3—O8⋯π inter­action [O8⋯*Cg*1 = 3.272 (6) Å, *Cg*1 is the centroid of the C1–C6 ring] and a C9=O4⋯π inter­action [O4⋯*Cg*2 = 3.552 (7) Å, *Cg*2 is the centroid of the C17–C22 ring].

## Synthesis and crystallization

In an oven-dried Schlenk reaction vessel, diisopropyl 2-[(tos­yloxy)imino]­malonate (185.7 mg, 0.5 mmol, 1.0 equiv.) was dissolved in anhydrous THF (5 ml) under Ar and cooled to 273 K on an ice bath. To the cooled solution, Et_3_N (1.0 mmol, 2.0 equiv.) and hydrazine (0.5 mmol, 1.0 equiv.) were added. The reaction mixture was allowed to stir at 273 K for 2 h. After full conversion as indicated by TLC, Et_3_N (1.0 mmol, 2.0 equiv.) and 4-nitro­benzoyl chloride (92.8 mg, 0.5 mmol, 1.0 equiv.) were added to the reaction mixture. After stirring the reaction mixture for 3 h at 273 K, a saturated NaHCO_3_ solution was added and the mixture was extracted with diethyl ether. The combined organic fractions were concentrated *in vacuo*. The crude product was purified using silica gel flash column chromatography to afford the title compound (90.0 mg, 51% yield). Crystals were obtained by dissolving the compound in a minimum amount of CH_2_Cl_2_ and layering with hexane at 295 K.

## Refinement

Crystal data, data collection and structure refinement are summarized in Table 1 ▸.

## Supplementary Material

Crystal structure: contains datablock(s) I. DOI: 10.1107/S2414314623001049/vm4057sup1.cif

Structure factors: contains datablock(s) I. DOI: 10.1107/S2414314623001049/vm4057Isup2.hkl

Supporting information file. DOI: 10.1107/S2414314623001049/vm4057Isup3.mol

Supporting information file. DOI: 10.1107/S2414314623001049/vm4057Isup4.cml

CCDC reference: 2240109

Additional supporting information:  crystallographic information; 3D view; checkCIF report

## Figures and Tables

**Table 1:** Experimental details

Crystal data	
Chemical formula	C_16_H_20_N_2_O_7_
*M*_r_	352.34
Crystal system, space group	Monoclinic, *C*2
Temperature (K)	296
*a*, *b*, *c* (Å)	38.408 (10), 5.1523 (13), 19.752 (5)
β (°)	115.341 (4)
*V* (Å^3^)	3532.7 (15)
*Z*	8
Radiation type	Mo *K*α
μ (mm^−1^)	0.11
Crystal size (mm)	0.50 × 0.08 × 0.05

Data collection	
Diffractometer	Bruker APEXII CCD
Absorption correction	Multi-scan (*SADABS*; Krause *et al.*, 2015 ▸)
*T*_min_, *T*_max_	0.534, 0.745
No. of measured, independent and observed [*I* > 2σ(*I*)] reflections	18724, 8693, 3977
*R*_int_	0.062
(sin θ/λ)_max_ (Å^−1^)	0.668

Refinement	
*R*[*F* ^2^ > 2σ(*F* ^2^)], *wR*(*F* ^2^), *S*	0.081, 0.276, 1.00
No. of reflections	8693
No. of parameters	459
No. of restraints	52
H-atom treatment	H-atom parameters constrained
Δρ_max_, Δρ_min_ (e Å^−3^)	0.49, −0.34
Absolute structure	Flack *x* determined using 1214 quotients [(*I* ^+^)−(*I* ^−^)]/[(*I* ^+^)+(*I* ^−^)] (Parsons *et al.*, 2013 ▸)
Absolute structure parameter	0.6 (10)

Computer programs: *APEX2* and *SAINT* (Bruker, 2016 ▸), *SHELXT2014/4* (Sheldrick, 2015*a* ▸), 4*SHELXL2018/4*3 (Sheldrick, 2015*b* ▸), and *SHELXTL* (Sheldrick, 2008 ▸).

## full crystallographic data

### Crystal data

**Table:**

C_16_H_20_N_2_O_7_	*F*(000) = 1488
*M**_r_* = 352.34	*D*_x_ = 1.325 Mg m^−^^3^
Monoclinic, *C*2	Mo *K*α radiation, λ = 0.71073 Å
*a* = 38.408 (10) Å	Cell parameters from 2123 reflections
*b* = 5.1523 (13) Å	θ = 2.3–19.0°
*c* = 19.752 (5) Å	µ = 0.11 mm^−^^1^
β = 115.341 (4)°	*T* = 296 K
*V* = 3532.7 (15) Å^3^	Needle, colourless
*Z* = 8	0.50 × 0.08 × 0.05 mm

### Data collection

**Table:**

Bruker APEXII CCD diffractometer	3977 reflections with *I* > 2σ(*I*)
φ and ω scans	*R*_int_ = 0.062
Absorption correction: multi-scan (SADABS; Krause *et al.*, 2015)	θ_max_ = 28.4°, θ_min_ = 2.0°
*T*_min_ = 0.534, *T*_max_ = 0.745	*h* = −50→50
18724 measured reflections	*k* = −6→6
8693 independent reflections	*l* = −26→26

### Refinement

**Table:**

Refinement on *F*^2^	Hydrogen site location: inferred from neighbouring sites
Least-squares matrix: full	H-atom parameters constrained
*R*[*F*^2^ > 2σ(*F*^2^)] = 0.081	*w* = 1/[σ^2^(*F*_o_^2^) + (0.1417*P*)^2^] where *P* = (*F*_o_^2^ + 2*F*_c_^2^)/3
*wR*(*F*^2^) = 0.276	(Δ/σ)_max_ < 0.001
*S* = 1.00	Δρ_max_ = 0.49 e Å^−^^3^
8693 reflections	Δρ_min_ = −0.34 e Å^−^^3^
459 parameters	Absolute structure: Flack *x* determined using 1214 quotients [(*I*^+^)-(*I*^-^)]/[(*I*^+^)+(*I*^-^)] (Parsons *et al.*, 2013)
52 restraints	Absolute structure parameter: 0.6 (10)

### Special details

**Table:**

Geometry. All esds (except the esd in the dihedral angle between two l.s. planes) are estimated using the full covariance matrix. The cell esds are taken into account individually in the estimation of esds in distances, angles and torsion angles; correlations between esds in cell parameters are only used when they are defined by crystal symmetry. An approximate (isotropic) treatment of cell esds is used for estimating esds involving l.s. planes.

### Fractional atomic coordinates and isotropic or equivalent isotropic displacement parameters (Å^2^)

**Table:**

	*x*	*y*	*z*	*U*_iso_*/*U*_eq_	
O1	0.88102 (19)	0.2244 (16)	0.6329 (5)	0.118 (2)	
O2	0.90133 (17)	0.5358 (18)	0.7072 (4)	0.128 (3)	
O3	0.71544 (12)	0.9400 (9)	0.6084 (3)	0.0712 (13)	
O4	0.67465 (15)	0.5799 (14)	0.6930 (3)	0.0944 (19)	
O5	0.61676 (14)	0.7265 (11)	0.6190 (3)	0.0753 (14)	
O6	0.63941 (14)	0.2095 (10)	0.4886 (3)	0.0837 (16)	
O7	0.59253 (12)	0.4686 (11)	0.4841 (3)	0.0795 (15)	
N1	0.87565 (19)	0.4161 (17)	0.6630 (4)	0.0811 (18)	
N2	0.69438 (14)	0.5333 (10)	0.5764 (3)	0.0555 (13)	
H2N2	0.700003	0.375915	0.570495	0.067*	
C1	0.83549 (19)	0.4910 (16)	0.6442 (4)	0.0641 (18)	
C2	0.8060 (2)	0.3469 (16)	0.5940 (4)	0.0681 (18)	
H2	0.810805	0.206553	0.569638	0.082*	
C3	0.76912 (18)	0.4137 (14)	0.5804 (3)	0.0577 (16)	
H3	0.748686	0.313843	0.547615	0.069*	
C4	0.76178 (16)	0.6251 (12)	0.6142 (3)	0.0500 (14)	
C5	0.79215 (18)	0.7752 (13)	0.6635 (4)	0.0599 (16)	
H5	0.787483	0.920965	0.686133	0.072*	
C6	0.82960 (18)	0.7034 (15)	0.6783 (4)	0.0682 (19)	
H6	0.850388	0.800012	0.711361	0.082*	
C7	0.72186 (18)	0.7100 (13)	0.6000 (3)	0.0551 (15)	
C8	0.65570 (17)	0.5975 (12)	0.5606 (4)	0.0555 (15)	
H8	0.649966	0.764484	0.534248	0.067*	
C9	0.65032 (19)	0.6329 (14)	0.6321 (4)	0.0604 (16)	
C10	0.6067 (3)	0.7816 (19)	0.6811 (4)	0.086 (3)	
H10	0.622571	0.675836	0.724629	0.103*	
C11	0.6145 (3)	1.065 (2)	0.6998 (6)	0.122 (4)	
H11A	0.641204	1.100776	0.713532	0.182*	
H11B	0.608425	1.107266	0.740821	0.182*	
H11C	0.598852	1.167367	0.656892	0.182*	
C12	0.5657 (3)	0.712 (2)	0.6551 (7)	0.120 (4)	
H12A	0.550242	0.819530	0.613445	0.181*	
H12B	0.558195	0.736768	0.695147	0.181*	
H12C	0.562101	0.532895	0.639884	0.181*	
C13	0.62848 (17)	0.3987 (13)	0.5080 (3)	0.0539 (15)	
C14	0.5632 (2)	0.3031 (18)	0.4300 (5)	0.089 (3)	
H14	0.574593	0.184705	0.406273	0.107*	
C15	0.5350 (4)	0.490 (3)	0.3721 (8)	0.156 (5)	
H15A	0.518994	0.570858	0.392476	0.235*	
H15B	0.519074	0.395818	0.327625	0.235*	
H15C	0.549129	0.621206	0.359918	0.235*	
C16	0.5450 (4)	0.157 (4)	0.4700 (9)	0.172 (6)	
H16A	0.564320	0.061675	0.510334	0.258*	
H16B	0.526439	0.038554	0.436017	0.258*	
H16C	0.532346	0.275346	0.489680	0.258*	
O8	0.80326 (17)	0.2861 (12)	0.7820 (3)	0.0891 (17)	
O9	0.81622 (17)	0.6196 (13)	0.8529 (4)	0.0951 (18)	
O10	0.66470 (15)	−0.0673 (10)	0.8979 (3)	0.0747 (13)	
O11	0.65449 (17)	0.6482 (13)	1.0333 (3)	0.0934 (18)	
O12	0.60149 (14)	0.4539 (13)	1.0243 (3)	0.0924 (18)	
O13	0.58449 (16)	0.4185 (15)	0.8261 (3)	0.103 (2)	
O14	0.56464 (13)	0.1570 (11)	0.8898 (3)	0.0754 (14)	
N3	0.79752 (15)	0.4276 (13)	0.8251 (3)	0.0664 (15)	
N4	0.65984 (14)	0.3465 (11)	0.9276 (3)	0.0595 (14)	
H4N4	0.668115	0.503086	0.930582	0.071*	
C17	0.76625 (18)	0.3554 (13)	0.8470 (4)	0.0572 (16)	
C18	0.7437 (2)	0.1486 (14)	0.8140 (4)	0.0645 (17)	
H18	0.747804	0.051632	0.778358	0.077*	
C19	0.71472 (18)	0.0839 (13)	0.8336 (4)	0.0585 (16)	
H19	0.699443	−0.060793	0.812472	0.070*	
C20	0.70813 (17)	0.2356 (12)	0.8855 (3)	0.0507 (14)	
C21	0.73146 (18)	0.4425 (14)	0.9176 (4)	0.0594 (16)	
H21	0.727253	0.542696	0.952543	0.071*	
C22	0.76095 (19)	0.5053 (15)	0.8993 (4)	0.0639 (17)	
H22	0.776969	0.645826	0.921640	0.077*	
C23	0.67578 (17)	0.1561 (13)	0.9041 (3)	0.0532 (15)	
C24	0.62899 (17)	0.2893 (13)	0.9479 (4)	0.0575 (16)	
H24	0.632877	0.115292	0.969983	0.069*	
C25	0.63007 (19)	0.4888 (14)	1.0067 (4)	0.0604 (16)	
C26	0.5982 (3)	0.642 (2)	1.0786 (5)	0.101 (3)	
H26	0.618275	0.775429	1.092635	0.121*	
C27	0.6031 (4)	0.483 (4)	1.1412 (8)	0.166 (5)	
H27A	0.583457	0.351385	1.125663	0.249*	
H27B	0.601108	0.587697	1.179561	0.249*	
H27C	0.627983	0.401613	1.160547	0.249*	
C28	0.5600 (4)	0.757 (3)	1.0432 (9)	0.163 (5)	
H28A	0.541281	0.622836	1.019716	0.245*	
H28B	0.559144	0.880016	1.006063	0.245*	
H28C	0.554309	0.842906	1.080354	0.245*	
C29	0.5902 (2)	0.2985 (14)	0.8801 (4)	0.0601 (16)	
C30	0.5250 (2)	0.1611 (18)	0.8325 (4)	0.080 (2)	
H30	0.522370	0.300077	0.796814	0.096*	
C31	0.5161 (5)	−0.083 (4)	0.7929 (10)	0.184 (6)	
H31A	0.515314	−0.218117	0.825734	0.277*	
H31B	0.535656	−0.122663	0.776323	0.277*	
H31C	0.491564	−0.071294	0.750378	0.277*	
C32	0.5000 (3)	0.216 (5)	0.8673 (8)	0.185 (8)	
H32A	0.473706	0.210829	0.830379	0.277*	
H32B	0.505665	0.386042	0.889470	0.277*	
H32C	0.503806	0.089532	0.905427	0.277*	

### Atomic displacement parameters (Å^2^)

**Table:**

	*U*^11^	*U*^22^	*U*^33^	*U*^12^	*U*^13^	*U*^23^
O1	0.083 (4)	0.128 (6)	0.150 (6)	0.017 (4)	0.058 (4)	−0.019 (5)
O2	0.050 (3)	0.177 (8)	0.134 (6)	0.000 (4)	0.019 (4)	−0.021 (6)
O3	0.059 (3)	0.056 (3)	0.094 (3)	0.000 (2)	0.029 (2)	−0.010 (3)
O4	0.073 (3)	0.142 (6)	0.064 (3)	0.034 (4)	0.025 (3)	0.015 (3)
O5	0.063 (3)	0.101 (4)	0.063 (3)	0.023 (3)	0.028 (2)	0.000 (3)
O6	0.063 (3)	0.064 (3)	0.103 (4)	0.005 (2)	0.015 (3)	−0.014 (3)
O7	0.041 (2)	0.100 (4)	0.090 (3)	−0.005 (3)	0.021 (2)	−0.031 (3)
N1	0.058 (4)	0.105 (6)	0.087 (4)	0.008 (4)	0.038 (4)	0.009 (4)
N2	0.045 (3)	0.047 (3)	0.074 (3)	−0.002 (2)	0.024 (3)	−0.001 (2)
C1	0.054 (4)	0.084 (5)	0.065 (4)	0.007 (4)	0.036 (3)	0.011 (4)
C2	0.064 (4)	0.075 (4)	0.072 (4)	−0.001 (4)	0.036 (4)	−0.008 (4)
C3	0.051 (3)	0.067 (4)	0.054 (3)	−0.007 (3)	0.021 (3)	−0.009 (3)
C4	0.050 (3)	0.053 (3)	0.047 (3)	−0.001 (3)	0.021 (3)	0.006 (3)
C5	0.056 (4)	0.056 (4)	0.065 (4)	−0.005 (3)	0.023 (3)	−0.012 (3)
C6	0.044 (4)	0.080 (5)	0.073 (4)	−0.014 (3)	0.018 (3)	−0.011 (4)
C7	0.055 (4)	0.057 (4)	0.049 (3)	−0.003 (3)	0.019 (3)	0.000 (3)
C8	0.053 (4)	0.051 (4)	0.063 (4)	0.004 (3)	0.025 (3)	0.002 (3)
C9	0.054 (4)	0.066 (4)	0.057 (4)	0.004 (3)	0.020 (3)	0.000 (3)
C10	0.083 (6)	0.116 (7)	0.067 (5)	0.034 (5)	0.039 (4)	−0.001 (5)
C11	0.136 (9)	0.121 (9)	0.097 (7)	0.020 (7)	0.039 (7)	−0.030 (7)
C12	0.096 (7)	0.151 (10)	0.145 (9)	0.020 (7)	0.081 (7)	0.011 (8)
C13	0.050 (4)	0.053 (4)	0.053 (3)	0.002 (3)	0.017 (3)	−0.002 (3)
C14	0.047 (4)	0.104 (6)	0.107 (6)	0.010 (4)	0.023 (4)	−0.036 (5)
C15	0.154 (5)	0.156 (5)	0.153 (5)	−0.004 (3)	0.060 (3)	0.004 (3)
C16	0.171 (6)	0.172 (6)	0.171 (6)	−0.009 (3)	0.071 (3)	0.002 (3)
O8	0.101 (4)	0.101 (4)	0.094 (4)	−0.011 (3)	0.070 (3)	−0.013 (3)
O9	0.094 (4)	0.094 (4)	0.120 (5)	−0.019 (4)	0.068 (4)	−0.005 (4)
O10	0.088 (3)	0.056 (3)	0.095 (3)	−0.010 (3)	0.054 (3)	−0.003 (3)
O11	0.102 (4)	0.113 (4)	0.090 (4)	−0.045 (4)	0.064 (3)	−0.032 (3)
O12	0.076 (3)	0.124 (5)	0.098 (4)	−0.021 (3)	0.057 (3)	−0.046 (4)
O13	0.082 (4)	0.141 (6)	0.083 (4)	−0.008 (4)	0.034 (3)	0.038 (4)
O14	0.060 (3)	0.092 (4)	0.072 (3)	−0.013 (3)	0.026 (3)	0.006 (3)
N3	0.057 (3)	0.071 (4)	0.077 (4)	−0.004 (3)	0.034 (3)	0.007 (3)
N4	0.055 (3)	0.052 (3)	0.081 (4)	0.000 (2)	0.038 (3)	0.003 (3)
C17	0.051 (3)	0.064 (4)	0.059 (4)	0.003 (3)	0.026 (3)	0.012 (3)
C18	0.072 (4)	0.064 (4)	0.065 (4)	−0.001 (4)	0.036 (3)	−0.009 (3)
C19	0.058 (4)	0.056 (4)	0.066 (4)	0.000 (3)	0.031 (3)	−0.010 (3)
C20	0.046 (3)	0.050 (3)	0.057 (3)	0.005 (3)	0.023 (3)	0.006 (3)
C21	0.058 (4)	0.064 (4)	0.062 (4)	−0.004 (3)	0.031 (3)	−0.008 (3)
C22	0.059 (4)	0.069 (4)	0.064 (4)	−0.008 (3)	0.026 (3)	−0.008 (3)
C23	0.055 (4)	0.052 (4)	0.057 (4)	0.000 (3)	0.029 (3)	0.002 (3)
C24	0.055 (4)	0.062 (4)	0.071 (4)	0.004 (3)	0.041 (3)	0.006 (3)
C25	0.057 (4)	0.068 (4)	0.061 (4)	−0.008 (4)	0.029 (3)	0.002 (3)
C26	0.101 (3)	0.104 (3)	0.101 (3)	−0.0013 (14)	0.0461 (17)	−0.0033 (14)
C27	0.169 (5)	0.166 (5)	0.162 (5)	0.011 (3)	0.070 (3)	−0.003 (3)
C28	0.164 (5)	0.164 (5)	0.162 (5)	0.009 (3)	0.071 (3)	−0.002 (3)
C29	0.067 (4)	0.067 (4)	0.058 (4)	0.006 (3)	0.038 (3)	0.007 (3)
C30	0.066 (5)	0.088 (5)	0.074 (5)	−0.007 (4)	0.020 (4)	−0.009 (4)
C31	0.182 (6)	0.182 (7)	0.185 (7)	0.003 (3)	0.075 (4)	−0.005 (3)
C32	0.087 (7)	0.32 (2)	0.153 (11)	0.015 (11)	0.056 (8)	−0.026 (15)

### Geometric parameters (Å, º)

**Table:**

O1—N1	1.216 (10)	O8—N3	1.210 (7)
O2—N1	1.174 (9)	O9—N3	1.207 (8)
O3—C7	1.236 (8)	O10—C23	1.215 (8)
O4—C9	1.197 (7)	O11—C25	1.186 (8)
O5—C9	1.294 (8)	O12—C25	1.297 (8)
O5—C10	1.463 (9)	O12—C26	1.491 (11)
O6—C13	1.188 (8)	O13—C29	1.170 (8)
O7—C13	1.304 (8)	O14—C29	1.299 (8)
O7—C14	1.452 (9)	O14—C30	1.459 (9)
N1—C1	1.475 (9)	N3—C17	1.488 (8)
N2—C7	1.318 (8)	N4—C23	1.340 (8)
N2—C8	1.420 (7)	N4—C24	1.434 (7)
N2—H2N2	0.8600	N4—H4N4	0.8600
C1—C6	1.354 (10)	C17—C18	1.353 (10)
C1—C2	1.363 (10)	C17—C22	1.373 (9)
C2—C3	1.368 (9)	C18—C19	1.367 (9)
C2—H2	0.9300	C18—H18	0.9300
C3—C4	1.368 (9)	C19—C20	1.395 (8)
C3—H3	0.9300	C19—H19	0.9300
C4—C5	1.391 (8)	C20—C21	1.362 (9)
C4—C7	1.501 (9)	C20—C23	1.496 (8)
C5—C6	1.389 (9)	C21—C22	1.367 (9)
C5—H5	0.9300	C21—H21	0.9300
C6—H6	0.9300	C22—H22	0.9300
C8—C13	1.514 (9)	C24—C29	1.520 (9)
C8—C9	1.525 (9)	C24—C25	1.538 (10)
C8—H8	0.9800	C24—H24	0.9800
C10—C12	1.475 (13)	C26—C27	1.430 (17)
C10—C11	1.504 (15)	C26—C28	1.454 (16)
C10—H10	0.9800	C26—H26	0.9800
C11—H11A	0.9600	C27—H27A	0.9600
C11—H11B	0.9600	C27—H27B	0.9600
C11—H11C	0.9600	C27—H27C	0.9600
C12—H12A	0.9600	C28—H28A	0.9600
C12—H12B	0.9600	C28—H28B	0.9600
C12—H12C	0.9600	C28—H28C	0.9600
C14—C16	1.465 (16)	C30—C32	1.427 (13)
C14—C15	1.534 (17)	C30—C31	1.44 (2)
C14—H14	0.9800	C30—H30	0.9800
C15—H15A	0.9600	C31—H31A	0.9600
C15—H15B	0.9600	C31—H31B	0.9600
C15—H15C	0.9600	C31—H31C	0.9600
C16—H16A	0.9600	C32—H32A	0.9600
C16—H16B	0.9600	C32—H32B	0.9600
C16—H16C	0.9600	C32—H32C	0.9600
			
C9—O5—C10	120.1 (5)	C25—O12—C26	116.4 (7)
C13—O7—C14	118.2 (6)	C29—O14—C30	118.8 (6)
O2—N1—O1	121.8 (8)	O9—N3—O8	124.2 (6)
O2—N1—C1	120.3 (8)	O9—N3—C17	118.0 (6)
O1—N1—C1	117.9 (8)	O8—N3—C17	117.8 (6)
C7—N2—C8	121.4 (5)	C23—N4—C24	120.1 (6)
C7—N2—H2N2	119.3	C23—N4—H4N4	120.0
C8—N2—H2N2	119.3	C24—N4—H4N4	120.0
C6—C1—C2	122.6 (6)	C18—C17—C22	122.4 (6)
C6—C1—N1	117.7 (7)	C18—C17—N3	119.1 (6)
C2—C1—N1	119.6 (7)	C22—C17—N3	118.5 (6)
C1—C2—C3	118.3 (7)	C17—C18—C19	119.1 (6)
C1—C2—H2	120.8	C17—C18—H18	120.5
C3—C2—H2	120.8	C19—C18—H18	120.5
C2—C3—C4	121.1 (6)	C18—C19—C20	119.8 (6)
C2—C3—H3	119.4	C18—C19—H19	120.1
C4—C3—H3	119.4	C20—C19—H19	120.1
C3—C4—C5	119.9 (6)	C21—C20—C19	119.3 (6)
C3—C4—C7	123.2 (6)	C21—C20—C23	123.4 (5)
C5—C4—C7	116.9 (6)	C19—C20—C23	117.3 (6)
C6—C5—C4	118.8 (6)	C20—C21—C22	121.1 (6)
C6—C5—H5	120.6	C20—C21—H21	119.4
C4—C5—H5	120.6	C22—C21—H21	119.4
C1—C6—C5	119.2 (6)	C21—C22—C17	118.2 (6)
C1—C6—H6	120.4	C21—C22—H22	120.9
C5—C6—H6	120.4	C17—C22—H22	120.9
O3—C7—N2	122.3 (6)	O10—C23—N4	122.4 (6)
O3—C7—C4	120.0 (6)	O10—C23—C20	121.8 (6)
N2—C7—C4	117.7 (6)	N4—C23—C20	115.8 (6)
N2—C8—C13	110.1 (5)	N4—C24—C29	111.4 (5)
N2—C8—C9	111.6 (5)	N4—C24—C25	108.4 (5)
C13—C8—C9	112.9 (5)	C29—C24—C25	110.1 (5)
N2—C8—H8	107.3	N4—C24—H24	109.0
C13—C8—H8	107.3	C29—C24—H24	109.0
C9—C8—H8	107.3	C25—C24—H24	109.0
O4—C9—O5	124.7 (6)	O11—C25—O12	125.2 (7)
O4—C9—C8	123.2 (6)	O11—C25—C24	123.9 (6)
O5—C9—C8	112.1 (5)	O12—C25—C24	110.9 (6)
O5—C10—C12	106.7 (7)	C27—C26—C28	112.4 (11)
O5—C10—C11	107.3 (8)	C27—C26—O12	103.2 (10)
C12—C10—C11	113.2 (9)	C28—C26—O12	107.3 (9)
O5—C10—H10	109.9	C27—C26—H26	111.2
C12—C10—H10	109.9	C28—C26—H26	111.2
C11—C10—H10	109.9	O12—C26—H26	111.2
C10—C11—H11A	109.5	C26—C27—H27A	109.5
C10—C11—H11B	109.5	C26—C27—H27B	109.5
H11A—C11—H11B	109.5	H27A—C27—H27B	109.5
C10—C11—H11C	109.5	C26—C27—H27C	109.5
H11A—C11—H11C	109.5	H27A—C27—H27C	109.5
H11B—C11—H11C	109.5	H27B—C27—H27C	109.5
C10—C12—H12A	109.5	C26—C28—H28A	109.5
C10—C12—H12B	109.5	C26—C28—H28B	109.5
H12A—C12—H12B	109.5	H28A—C28—H28B	109.5
C10—C12—H12C	109.5	C26—C28—H28C	109.5
H12A—C12—H12C	109.5	H28A—C28—H28C	109.5
H12B—C12—H12C	109.5	H28B—C28—H28C	109.5
O6—C13—O7	125.1 (6)	O13—C29—O14	124.9 (7)
O6—C13—C8	122.7 (6)	O13—C29—C24	123.3 (7)
O7—C13—C8	112.0 (6)	O14—C29—C24	111.8 (5)
O7—C14—C16	107.7 (9)	C32—C30—C31	112.6 (13)
O7—C14—C15	104.9 (9)	C32—C30—O14	108.9 (8)
C16—C14—C15	112.2 (9)	C31—C30—O14	109.4 (9)
O7—C14—H14	110.6	C32—C30—H30	108.6
C16—C14—H14	110.6	C31—C30—H30	108.6
C15—C14—H14	110.6	O14—C30—H30	108.6
C14—C15—H15A	109.5	C30—C31—H31A	109.5
C14—C15—H15B	109.5	C30—C31—H31B	109.5
H15A—C15—H15B	109.5	H31A—C31—H31B	109.5
C14—C15—H15C	109.5	C30—C31—H31C	109.5
H15A—C15—H15C	109.5	H31A—C31—H31C	109.5
H15B—C15—H15C	109.5	H31B—C31—H31C	109.5
C14—C16—H16A	109.5	C30—C32—H32A	109.5
C14—C16—H16B	109.5	C30—C32—H32B	109.5
H16A—C16—H16B	109.5	H32A—C32—H32B	109.5
C14—C16—H16C	109.5	C30—C32—H32C	109.5
H16A—C16—H16C	109.5	H32A—C32—H32C	109.5
H16B—C16—H16C	109.5	H32B—C32—H32C	109.5
